# Pulmonary Mucus Gland Adenomas: Are They Always of Endobronchial Localization?

**DOI:** 10.1155/2013/239173

**Published:** 2013-03-04

**Authors:** Georgia Karpathiou, Efthimios Sivridis, Dimitrios Mikroulis, Marios Froudarakis, Alexandra Giatromanolaki

**Affiliations:** ^1^Department of Pathology, Medical School, Democritus University of Thrace, 68100 Alexandroupolis, Greece; ^2^Department of Cardiothoracic Surgery, Medical School, Democritus University of Thrace, 68100 Alexandroupolis, Greece; ^3^Department of Pneumonology, Medical School, Democritus University of Thrace, 68100 Alexandroupolis, Greece

## Abstract

Mucus gland adenoma is an extremely rare benign lung tumor, presumed to arise from the bronchial mucus glands; it is a TTF-1 negative tumor, centrally located, causing the clinical manifestations of obstruction. We report a TTF-1 negative mucus gland adenoma, arising into the medial bronchopulmonary segment, lacking any relation to a bronchus.

## 1. Introduction

Mucus gland adenoma of the lung represents a genuine adenoma of salivary gland type, characterized microscopically by mucus-filled cysts, acini and glandular structures, lined by a single layer of epithelial cells having not, or only exceptionally, the features of cytological atypia [7]. It is an extremely rare tumor, occurring in all ages, causing signs and symptoms of obstruction, given that it arises from the seromucous glands of the submucosa as a proximal exophytic mass [2]. We report a case of this unusual pulmonary tumor, originating not inside a bronchus, but rather from lung parenchyma, raising the question of this tumor's histogenesis and introducing differential diagnostic problems with peripheral lung tumors.

## 2. Case Report

**Figure 1:**
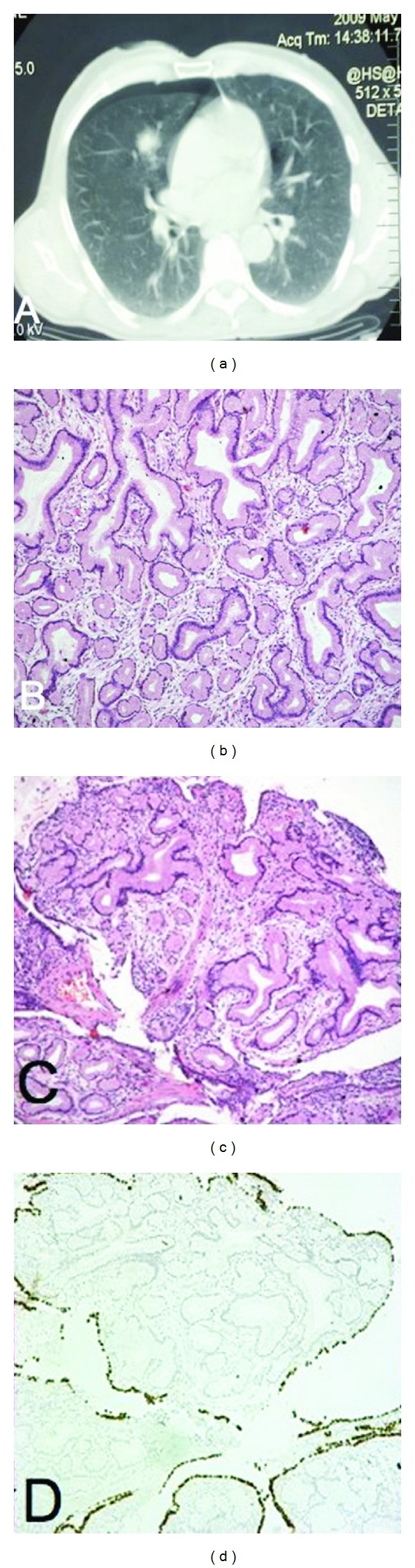
(a) Chest computed tomography during transthoracic needle biopsy, showing a parenchymal lesion of the middle lobe. (b) Mucus gland adenoma (H&E, ×200). (c) and (d) H&E and TTF-1 staining of the adenoma (×100).

A 59-year-old male, current smoker of 90 pack per year, underwent a chest radiograph, after an episode of acute bronchitis, which revealed a coin lesion in the right lung. Chest computed tomography confirmed the presence of a parenchymal lesion in the middle lobe (Figure 1(a)). Fiberoptic bronchoscopy revealed no intraluminal lesion, whereas transthoracic needle biopsy of the mass was not diagnostic, consisting purely of normal lung parenchyma. The presumptive clinical diagnosis was that of a pulmonary malignancy and the patient underwent right middle lobectomy and mediastinal lymph node sampling.

Macroscopic examination of the resected specimen revealed a whitish solid mass, 1.4 cm in maximum diameter, within the lung parenchyma of the medial bronchopulmonary segment. Microscopic examination of the resected specimen showed an area of neoplastic mucus glands lined by a single layer of tall columnar cells, with basal located nuclei and abundant mucus-filled supranuclear cytoplasm (Figures 1(b) and 1(c)). There were no cytological atypias or mitotic figures. Some of the gland lumens also contained mucus, PAS positive, while the overlying epithelium was unremarkable. An origin from a bronchus could not be confirmed despite examining multiple sections [2]. Immunohistochemical investigation for thyroid transcription factor-1 (TTF-1) was strongly positive for the overlying epithelium, but not for the neoplastic glands, which remained unreactive (Figure 1(d)). All mediastinal lymph nodes excised were free of disease and so did the patient 25 months after the operation.

## 3. Discussion

Mucus gland adenoma is a rare disease of the bronchial tree. It occurs proximally as an intraluminal tumor, 0.6 cm [1] to 6.8 cm [2] in diameter, most commonly at the age of 50 years [2]. No parenchymal location for such a tumor has been reported to date and the only mucus gland adenoma described peripherally [3] was, in fact, an acinar lesion arising from a dilated bronchus, covered by pseudostratified ciliated columnar epithelium [3]. This makes the adenoma presented here extremely unusual in regard to its localization inside lung parenchyma, with lack of any bronchial relation.

The clinical manifestations of the tumor include hemoptysis, cough, dyspnoea, and wheezing, often complicated with pneumonia [2, 4, 5], while imaging procedures reveal a coin lesion on radiography and a well-defined intraluminal mass at CT scans [6]. Mucus gland adenoma of our patient was a chance radiological finding, while examining an episode of acute bronchitis. 

Macroscopically, mucus gland adenoma appears as a white, smooth, and shiny tumor mass, with a cut surface, partly solid and partly cystic, with the cystic change being the predominant feature [2]. Unexpectedly, the tumor of our patient appeared smooth and solid, without cystic changes. Microscopic examination of these neoplasms reveals multiple mucus-filled acini and cysts, with or without small papillary formations lined by a tall columnar or cuboidal epithelium, admixed with goblet cells, oncocytes or clear cells, having no atypia or mitoses [7]. The described parenchymal adenoma consisted of mucus glands with occasional cyst formation and small acini.

Immunohistochemically, a bronchial mucus gland adenoma usually expresses high molecular weight keratins, but it is negative for thyroid transcription factor-1 (TTF-1) [1]—a protein with a crucial role in lung development and, thereafter, in lung pulmonary function [8]. In normal mature lung tissue, TTF-1 expression is restricted to type I and type II pneumocytes [8, 9] and the epithelium of small-sized bronchioles [8]; more specifically, TTF-1 protein is expressed throughout the terminal respiratory unit (TRU), representing a lineage marker for TRU [8]. No other cells, including those of the mucoserous glands or the ciliated and mucinous cells of the bronchial and bronchiolar epithelium, show such a positive reaction [8, 9]. This was also confirmed in our study, where the adenoma was negative for TTF-1, whilst, as it was expected, the overlying epithelium clearly expressed TTF-1 antigen. These findings suggest that an adenoma with morphologic features of mucus gland adenoma can indeed arise peripherally, probably after glandular mucinous metaplasia of the lining epithelium.

The differential diagnosis of a mucus gland adenoma includes the malignant lesions of adenocarcinoma and low-grade mucoepidermoid carcinoma, as well as the benign adenomatous lesions of glandular papilloma, papillary adenoma, alveolar cell adenoma, and mucinous cystadenoma. An adenocarcinoma shows the typical features of malignancy, such as cytological atypia, mitoses, and an infiltrative growth pattern, whilst a low-grade mucoepidermoid carcinoma, apart from mucus-secreting cells, it contains squamoid cells and an intermediate type of oval cells [2, 7]. The glandular papilloma has an endobronchial growth pattern and typical fibrovascular cores, lined by ciliated or nonciliated columnar cells and a varying proportion of cuboidal and goblet cells [10]. Mucinous cystadenomas, papillary adenomas, and alveolar cell adenomas are parenchymal lesions and, as such, are entering the differential diagnosis with our peripheral nonendobronchial case. A papillary adenoma, however, consists of fibrovascular cores lined by cuboidal or columnar epithelium and it is positive for TTF-1, whereas an alveolar adenoma containing TTF-1 positive cells shows cystic-like spaces lined by cuboidal cells [7]. A mucinous cystadenoma, on the other hand, is a true mucin-filled cyst lined by mucous epithelium, with variable expression of TTF-1 antigen [11].

Surgical airway resection, sparing lung parenchyma, is the treatment of choice for centrally located adenomas [4]. In case of total bronchial obstruction, with subsequent severe parenchymal lesions, lobectomy is preferred [4]. Bronchoscopic resection is another option reserved for patients with comorbidities. However, intraluminal treatment does not confirm the resection margins [4]. In our case, lobectomy was performed, due to the peripheral localization of the tumor and the high suspicion of malignancy.

To conclude, we describe the rare case of a mucus gland adenoma which, rather than having the typical intraluminal localization, arising from lung parenchyma causing differential diagnostic problems and raising the question of histogenesis.
